# Hyperreflective foci in OCT image as a biomarker of poor prognosis in diabetic macular edema patients treating with Conbercept in China

**DOI:** 10.1186/s12886-019-1168-0

**Published:** 2019-07-23

**Authors:** Shulin Liu, Desai Wang, Fei Chen, Xuedong Zhang

**Affiliations:** 1 The First Affiliated Hospital of Chongqing Medical University, Chongqing Key Laboratory of Ophthalmology and Chongqing Eye Institute, 1 You Yi Road, Yu Zhong District, Chongqing, 400016 People’s Republic of China; 2 Ophthalmology Department, The people’s Hospital of BiShan District of Chongqing City, Chongqing, People’s Republic of China

**Keywords:** Hyperreflective foci, Diabetic macular edema, Anti-VEGF, Conbercept

## Abstract

**Purpose:**

To investigate the dynamic changes of hyperreflective foci (HF) in diabetic macular edema (DME) patients during the intravitreal Conbercept treatment in China.

**Methods:**

DME Patients receiving intravitreal Conbercept (IVC) injections during the year 2016–2017 were retrospectively investigated. Thirteen patients (26 eyes) were recruited in this study. They received IVC once a month for 3 consecutive months. The number and location of HFs, the best-corrected visual acuity (BCVA) and central macular thickness (CMT) at each visit were analyzed and compared.

**Results:**

After the first injection, BCVA (LogMAR) was increased from 0.75 ± 0.48 to 0.43 ± 0.24 (*p* < 0.05), CMT improved from 575.9 ± 191.9 to 388.2 ± 198.5 μm (*p* = 0.014). However, the BCVA and CMT had no statistical difference after the second and third injection as compared with those after the first injection respectively. The baseline number of HFs was 5.39 ± 4.24, 5.15 ± 5.17 and 0.88 ± 1.90 in the inner retinal, outer retinal and subretinal layer respectively. The number of HFs in these three retinal layers decreased significantly after the first injection (*p* = 0.0045, *p* < 0.0001 and *p* = 0.0045, respectively). However, after the second injection, only the number of HFs in the inner retinal layer experienced a further decrease. After the third injection, no statistically significant HFs changes was observed in each retinal layers. Correlation analysis showed that there was a positive significant correlation between the baseline number of HFs in the inner retina, outer retina, subretina and final BCVA (r = 0.571, *p* = 0.002; r = 0.464, *p* = 0.017; r = 0.405, *p* = 0.04 respectively). There was also a significant positive correlation between outer retinal HFs reduction, total retinal HFs reduction and increase of BCVA (r = 0.40, *p* = 0.043 and r = 0.393, *p* = 0.04 respectively).

There were no severe ocular adverse reactions or systemic adverse events.

**Conclusions:**

Conbercept is effective and safe in the treatment of DME. HFs can act as a biomarker of poor final visual outcome.

## Introduction

Diabetic macular edema (DME) is a vision-threatening microvascular complication of diabetic retinopathy. It can occur at any stage of diabetic retinopathy and is the major cause of central visual loss in diabetic patients [1]. Anti-VEGF has now become the first line treatment regimen of DME for its excellent visual and anatomic improvement [2].

Conbercept (KH902; Chengdu Kanghong Biotech Co, Ltd., Sichuan, China) is a new member of anti-VEGF drug. Similar to aflibercept, Conbercept, with a molecular weight of 143 kDa, consisting of the binding domains of VEGF receptor 1 and VEGF receptor 2 fused to the Fc portion of human immunoglobulin G1 [3]. It can bind all isoforms of VEGF-A, placental growth factor, as well as VEGF-B with high affinity. Intravitreal Conbercept injection (IVC) has been demonstrated to be successful in the treatment of age-related macular degeneration [4], polypoidal choroidal vasculopathy [5], retinopathy of premature [6], choroidal neovascularization [7] and macular edema resulted from branch retinal vein occlusion [8]. In previous published papers, Conbercept has also been demonstrated to be effective in treating DME [9–11].

Retinal hyperreflective foci (HF) is defined as small discrete, well-circumscribed, dot-shaped lesion, with equal or greater reflectivity than the retinal pigment epithelium (RPE) band on spectral domain optical coherence tomography (SD-OCT) [12]. Recent studies suggested that HFs may predict the final visual outcome in macular edema patients treated with anti-VEGF agents [13–15]. In our clinic, we usually use 3 monthly Conbercept injection + PRN regiment to treat DME, however, the changes of HFs was not assessed in previous published works using this treatment strategy. Therefore, the aim of this study is to analyze the dynamic changes of HFs in DME patients treated with Conbercept, and to explore the relationship between HFs and the BCVA of DME patients.

## Methods

### Patients

This was a retrospective study and adhered to the tenets of the Declaration of Helsinki and approved by the Medical Ethics Committee of Chongqing medical university. At our clinic, all the DME patients were explained about different treatment strategy such as anti-VEGF intraocular injection (Lucentis and Conbercept, which were available in our clinic), and grid laser. We explained in detail how these treatment works and the risk and benefits the patients would get. The grid laser treatment was covered by medical insurance and the others two was not. Lucentis costed 1000 USD and Conbercept costed 700 USD for each injection. Patients who chose to be treated only with IVC were recruited between January 2016 and December 2017 at the first affiliated hospital of Chongqing medical university. Written consents were obtained from all the recruited patients before every injection.

Inclusion criteria were as follows: age ≥ 18 years and central macular thickness (CMT) ≥300 μm. Exclusion criteria were as follows: history or existence of other ocular disease that have an adverse effect on BCVA such as glaucoma, uveitis, vitreoretinal interface abnormalities and cataract, existence of any ocular disease that will cause poor-quality OCT images, history of vitreous surgery, macular ischemia diagnosed by fundus fluorescein angiography (FFA), intravitreal injection of anti-VEGF or steroids within 3 months, laser panphotocoagulation or macular grid laser within 4 months, high risk proliferative diabetic retinopathy and uncontrolled hypertension or recent cardiovascular event.

All the patients received 0.05 ml Conbercept monthly injection for a consecutive 3 months. They underwent a complete ophthalmologic examination, including BCVA, slit-lamp, indirect fundus examination and SD-OCT (Heidelberg Engineering, Heidelberg, Germany) at every visit. At baseline, FFA was also performed. As Conbercept has not been included in the medical insurance for treating DME in China, the patients has to pay a large sum of money. Therefore, most of the patients can just finish the first 3 injections, and that’s the reason we only analyzed the BCVA and CMT changes for the first 3 injections.

### HFs counting methods

The method used for counting HFs has been reported in several previous studies [16–18]. First, a horizontal B-scan passing through the fovea was taken by SD-OCT in all the patients at baseline and each visit. The number of HFs within the central 1 mm of the fovea were determined by one experienced masked grader (SL). We classified HFs into 3 groups according to their positions in the retinal layers: 1) In the outer retinal layers [from the external limiting membrane (ELM) to the photoreceptors], 2) In the inner retinal layers [from the internal limiting membrane (ILM) to the outer nuclear layer (ONL)], 3) Under RPE.

### Statistical analysis

All statistical analyses were carried out by version 22.0 SPSS statistical software (SPSS, Inc., Chicago, IL. USA). All the data was presented as mean ± SD. The BCVA was converted to a logarithm of the minimum angle of resolution (logMAR). The mean BCVA, HFs number and CMT between baseline and 1, 2 and 3 months were compared using one-way ANOVA. The post hoc test following ANOVA was Tukey’s test. Then, Pearson correlation analysis was used to analysis the correlation between the number of HFs and BCVA. *P* <0.05 was considered statistically significant.

## Results

### Characteristics of patients

A total of 26 eyes (13 patients) were included in this study including 76.9% males. All of these patients were Chinese and suffered from type 2 diabetes. The average age was 53.9 (rang 34–69).

At baseline, there were 2 eyes (7.7%) with mild nonproliferative diabetic retinopathy (NPDR), 7 eyes (26.9%) with moderate NPDR, 10 eyes (38.5%) with severe NPDR and 7 eyes (26.9%) with proliferative diabetic retinopathy (PDR). Fifteen eyes (57.7%) have been previously treated with PRP. All of them have never been treated with anti-VEGF drugs. The mean disease duration of DME before Conbercept treatment was 5.8 months (range 2–13, median 4).

### BCVA and CMT changes after Conbercept treatment

**Fig. 1:**
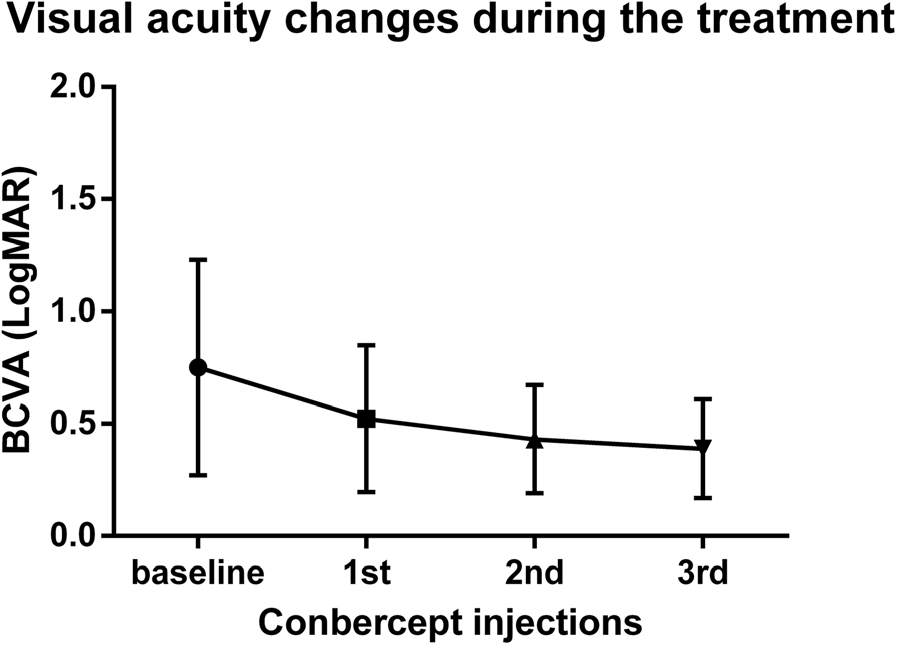
BCVA changes during the treatment of DME patients

The baseline BCVA (LogMAR) was 0.75 ± 0.48 and was improved to 0.52 ± 0.33 (*p* = 0.002), 0.43 ± 0.24 (*p* = 0.0001) and 0.39 ± 0.22 (*p* = 0.0002) at months 1, 2 and 3, respectively (Fig. 1). The BCVA at month 3 was significantly better than that at month 1 (*p* = 0.0033), however, the BCVA at month 2 had no statistical difference from that at month 1.

**Fig. 2:**
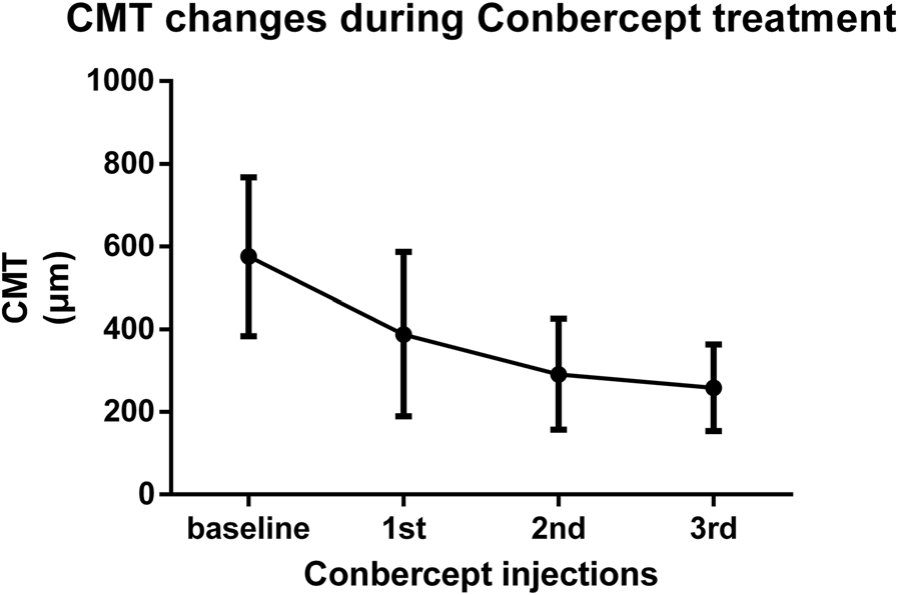
The changes of CMT during the Conbercept treatment

The baseline CMT was 575.9 ± 191.9 μm and decreased to 388.2 ± 198.5 μm (*p* < 0.0001), 291.5 ± 134.5 μm (*p* < 0.0001), and 258.6 ± 105.1 μm (p < 0.0001) at month 1, 2 and 3, respectively (Fig. 2). The CMT at month 2 was significantly lower than that at month 1 (*p* = 0.0028), however, the CMT at month 3 had no statistical difference from that in month 2.

### HFs in different retinal layers and its correlation with BCVA and CMT

**Fig. 3:**
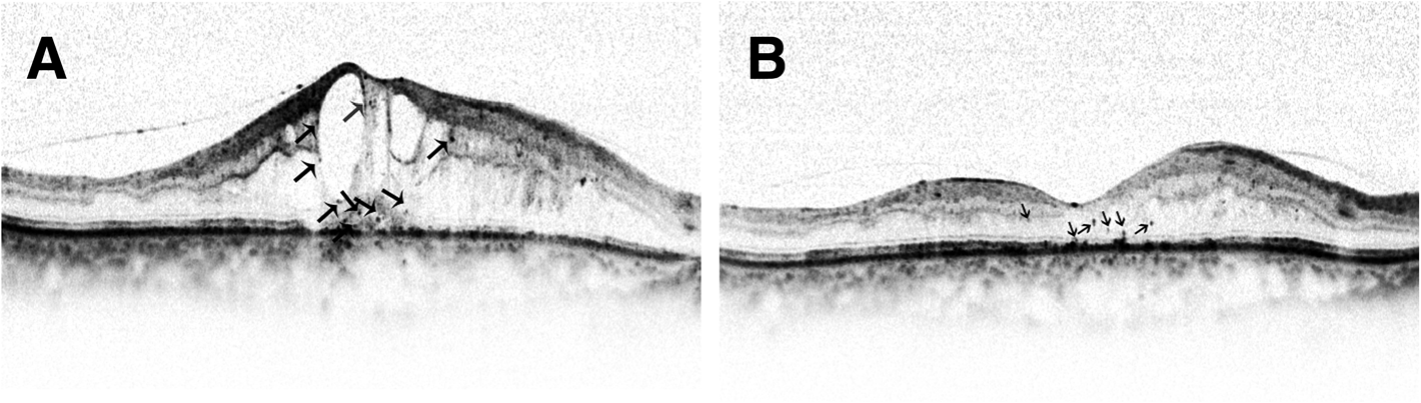
The HFs in the retinal layers before (**a**) and after (**b**) Conbercept treatment. HFs decreased after anti-VEGF treatment. Black arrows indicate hyperreflective foci

At baseline, HFs located in every retinal layers, but mainly in inner and outer retinal layers (Fig. 3a). The number of HFs was 5.4 ± 4.2 in inner retina, and 5.2 ± 5.2 in outer retina. There was no statistical difference concerning HFs numbers between these two layers. However, the number of HFs was only 0.88 ± 1.9 in the subretinal space, which was much lower than that of inner and outer retina (*p* = 0.0004 and *p* = 0.0008 respectively).

Correlation analysis was performed to evaluate the association of baseline and final BCVA (LogMAR) with baseline HFs in different retinal layers. The results showed that the baseline number of HFs in the outer retina was positively correlated with baseline BCVA (r = 0.42, *p* = 0.034). There was also a positive significant correlation between the baseline number of HFs in the inner retina, outer retina, subretina and final BCVA (r = 0.571, *p* = 0.002; r = 0.464, *p* = 0.017; r = 0.405, *p* = 0.04 respectively). Correlation analysis was also performed to evaluate the association of changes of HFs with changes of CMT and changes in BCVA (LogMAR). There was a significant positive correlation between inner retinal HFs reduction, total retinal HFs reduction and decrease of CMT (r = 0.422, *p* = 0.032 and r = 0.429, *p* = 0.029 respectively). There was also a significant positive correlation between outer retinal HFs reduction, total retinal HFs reduction and increase of BCVA (r = 0.40, *p* = 0.043 and r = 0.393, p = 0.04 respectively).

### Changes of HFs before and after Conbercept treatment

We analyzed the changes of HFs after Conbercept injection. At the baseline, the numbers of HFs in the inner retinal layer was 5.39 ± 4.24, it then decreased to 3.19 ± 2.15 (*p* = 0.0045) after the first Conbercept injection. After the second injection, it continuously decreased to 2.19 ± 2.00 (*p* = 0.0019). After the third injection, however, the number of HFs showed no statistical difference from that at month 2 (*p* = 0.447).

In the outer retinal layer, the numbers of HFs was 5.15 ± 5.17 at the baseline, it decreased to 3.35 ± 4.40 (*p*<0.0001) after the first Conbercept injection. After the second and third injection, however, there was no further significant decrease of HFs numbers.

**Fig. 4:**
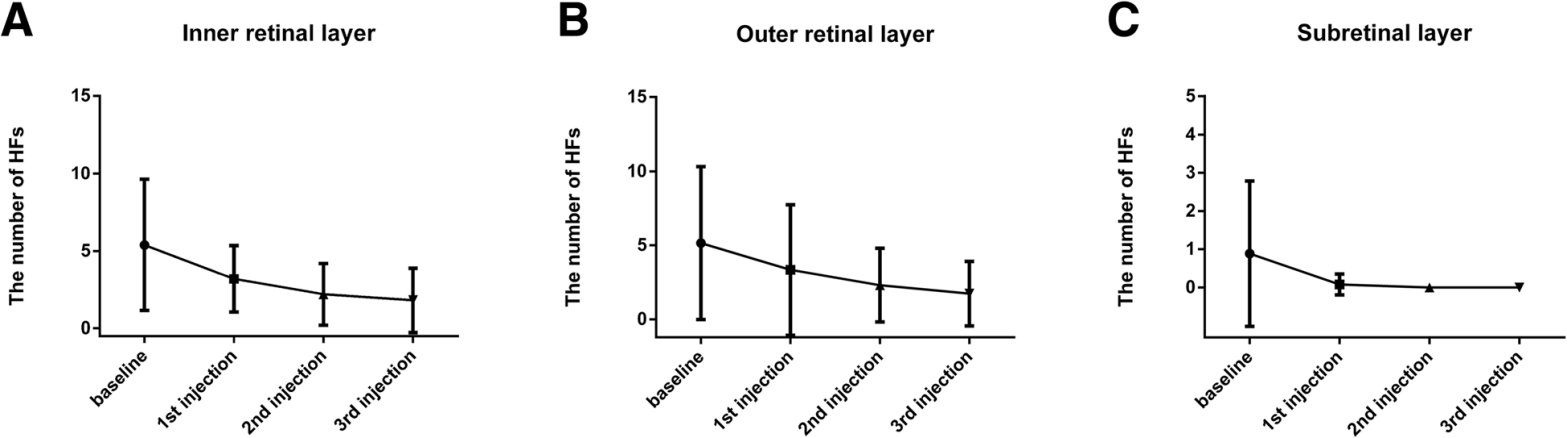
Changes of the number of HFs before and after anti-VEGF treatment. **a** Changes of the number of HFs in the inner retinal layer. **b** Changes of the number of HFs in the outer retinal layer. **c** Changes of the number of HFs in the subretinal layer

In the subretinal layer, the numbers of HFs was 0.88 ± 1.90 at the baseline, it then decreased to 0.08 ± 0.27 (*p* = 0.0045) after the first Conbercept injection. After the second injection, there was no subretinal HFs present, and it remained till month 3 (Fig. 4).

### Adverse events

Conbercept injections were relatively safe. No related serious adverse events and no endophthalmitis were reported.

## Discussion

This study characterizes the effectiveness of Conbercept in treating DME patients. The CMT decreased and visual acuity increased after treatment. Moreover, the reduction of the numbers of HFs is associated with the effect of Conbercept. Therefore, the number of HFs may be a predictive factor of poor final BCVA in DME patients treated with Conbercept.

Recently, HFs, which are discrete, focal, hyperreflective lesions, are observed in some retinal diseases, such as AMD, RVO, central serous choroidoretinopathy, Stargardt disease, retinitis pigmentosa as well as DR [15, 19–25]. Many theories have attempted to explain the pathophysiology of HFs, but they are still not fully understood. It is suggested that retinal inflammation will activate microglia cells and induce them to swell as well as spread to all retinal layers to become HFs in AMD [22]. In DR, HFs was thought to be small intraretinal proteins or lipid deposits which form after inner blood-retinal barrier breakdown, and they may be the precursor of hard exudates [13, 19]. Other theories suggested that HFs can occur even before the development of DR and represent neurodegerative process [26]. Previous studies described the migration of HF from the inner to the outer retina layers during the DR progression [27, 28]. Our results showed that HF was found in every retinal layers, but mainly located in the inner and outer retinal layer.

Vascular endothelial growth factor (VEGF) is believed to be the main driving force for the development and progression of DME [29]. Anti-VEGF agents have been found to reduce hyperpermeability through binding VEGF. Recent studies showed that after anti-VEGF treatment, retinal vessels hyperpermeability improved and the number of HF decreased in DME patients [14, 30]. To our knowledge, this is the first study to investigate the effect of Conbercept on the number of HFs over time. Our study, consistent with previous ones, demonstrated a significant reduction in HFs number after the first anti-VEGF treatment in patients with DME. This suggested that hyperpermeability with subsequent lipoprotein extravasation participate in the pathogenesis of HFs in DME patients. As Conbercept has been found to inhibit the breakdown of blood-retinal barrier in diabetic rats [31], we speculate that it may alleviate the damage of blood-retinal barrier in DME patients, and the underlying mechanism need to be investigated further. However, only in inner retinal layer, the HFs had significant reduction after the second anti-VEGF treatment. After the third anti-VEGF injection, HFs has no significant reduction in all of the layers.

In this study, we found that CMT is reduced after Conbercept treatment. Also, there was a significant reduction of HF in all retinal layers and improvement in BCVA. This further confirmed that Conbercept can improve the state of macula both anatomically and functionally. In line with previous studies, the number of HFs in the different retinal layers were associated with final BCVA in our study [14, 32], suggesting HF is a potential biomarker of poor outcome of DME. We also found that the inner retinal HFs reduction and total retinal HFs reduction had a moderate correlation with the final change of CMT. And the outer retinal HFs reduction and total retinal HFs reduction had a moderate correlation with the final change of BCVA. Therefore, HFs may be a reliable biomarker of individual response to Conbercept treatment in DME patients. We believe that HFs at baseline results from the disruption of blood–retinal barrier and can reflect the disease activity. Thus, we could predict that more HFs at baseline suggest a more active DME, thus will result in a worse final BCVA.

However, it should also be noted that this study has some limitations. First, it is a retrospective study, therefore, selection biases existed. Furthermore, it contained a relatively small sample size and short follow-up period. Notwithstanding these limitation, this investigation does determine the HFs as an affecting factor in Conbercept treatment of DME. In the future, randomized controlled trial can provide a deeper understanding of the influence of Conbercept on the changes of HFs.

In conclusion, this study demonstrates that Conbercept is effective in treating DME and baseline HFs numbers can be a biomarker of poor final visual outcome. Further researches are needed to focus on simplify of measuring HFs numbers in DME patients to guide the treatment.

## Data Availability

All the data were included in the manuscript.

## Abbreviations

BCVA: Best-corrected visual acuity
CMT: Central macular thickness
DME: Diabetic macular edema
ELM: External limiting membrane
HF: Hyperreflective foci
ILM: Internal limiting membrane
NPDR: Nonproliferative diabetic retinopathy
ONL: Outer nuclear layer
PDR: Proliferative diabetic retinopathy

## References

[1] Das A, McGuire P, Rangasamy S (2015). Diabetic macular edema: pathophysiology and novel therapeutic targets. Ophthalmology.

[2] Stewart M (2014). Anti-VEGF therapy for diabetic macular edema. Curr Diab Rep.

[3] Zhang M, Yu D, Yang C, Xia Q, Li W, Liu B, Li H (2009). The pharmacology study of a new recombinant human VEGF receptor-fc fusion protein on experimental choroidal neovascularization. Pharm Res.

[4] Li X, Xu G, Wang Y, Xu X, Liu X, Tang S, Zhang F, Zhang J, Tang L, Wu Q (2014). Safety and efficacy of conbercept in neovascular age-related macular degeneration: results from a 12-month randomized phase 2 study: AURORA study. Ophthalmology.

[5] Huang Z, Ding Q, Yan M, Lian H, Chen Z, Chen X, Song Y (2018). Short-term efficacy of CONBERCEPT and RANIBIZUMAB for POLYPOIDAL choroidal vasculopathy.

[6] Jin E, Yin H, Li X, Zhao M (2017). Short-term outcomes after intravitreal injections of CONBERCEPT versus RANIBIZUMAB for the treatment of retinopathy of prematurity.

[7] Peng Y, Zhang X, Mi L, Liu B, Zuo C, Li M, Wen F (2017). Efficacy and safety of conbercept as a primary treatment for choroidal neovascularization secondary to punctate inner choroidopathy. BMC Ophthalmol.

[8] Li F, Sun M, Guo J, Ma A, Zhao B (2017). Comparison of Conbercept with Ranibizumab for the treatment of macular edema secondary to branch retinal vein occlusion. Curr Eye Res.

[9] Li F, Zhang L, Wang Y, Xu W, Jiao W, Ma A, Zhao B (2018). One-year outcome of Conbercept therapy for diabetic macular edema. Curr Eye Res.

[10] Xu Y, Qu Y, Suo Y, Gao J, Chen X, Liu K, Xu X (2019). Correlation of retinal layer changes with vision gain in diabetic macular edema during conbercept treatment. BMC Ophthalmol.

[11] Xu Y, Rong A, Xu W, Niu Y, Wang Z (2017). Comparison of 12-month therapeutic effect of conbercept and ranibizumab for diabetic macular edema: a real-life clinical practice study. BMC Ophthalmol.

[12] Lee H, Lee J, Chung H, Kim HC (2016). Baseline spectral domain optical coherence tomographic HYPERREFLECTIVE foci as a predictor of visual outcome and recurrence for central serous CHORIORETINOPATHY. Retina.

[13] Nishijima K, Murakami T, Hirashima T, Uji A, Akagi T, Horii T, Ueda-Arakawa N, Muraoka Y, Yoshimura N (2014). Hyperreflective foci in outer retina predictive of photoreceptor damage and poor vision after vitrectomy for diabetic macular edema. Retina.

[14] Chatziralli IP, Sergentanis TN, Sivaprasad S (2016). HYPERREFLECTIVE foci as an independent visual outcome predictor in macular edema due to retinal vascular diseases treated with intravitreal dexamethasone or RANIBIZUMAB. Retina.

[15] De Benedetto U, Sacconi R, Pierro L, Lattanzio R, Bandello F (2015). Optical coherence tomographic hyperreflective foci in early stages of diabetic retinopathy. Retina.

[16] Nagasaka Y, Ito Y, Ueno S, Terasaki H. Number of Hyperreflective Foci in the Outer Retina Correlates with Inflammation and Photoreceptor Degeneration in Retinitis Pigmentosa. Ophthalmol Retina. 2018;2(7):726-34. 10.1016/j.oret.2017.07.020 31047383

[17] Framme C, Schweizer P, Imesch M, Wolf S, Wolf-Schnurrbusch U (2012). Behavior of SD-OCT-detected hyperreflective foci in the retina of anti-VEGF-treated patients with diabetic macular edema. Invest Ophthalmol Vis Sci.

[18] Mo B, Zhou HY, Jiao X, Zhang F (2017). Evaluation of hyperreflective foci as a prognostic factor of visual outcome in retinal vein occlusion. Int J Ophthalmol.

[19] Bolz M, Schmidt-Erfurth U, Deak G, Mylonas G, Kriechbaum K, Scholda C (2009). Optical coherence tomographic hyperreflective foci: a morphologic sign of lipid extravasation in diabetic macular edema. Ophthalmology.

[20] Chen KC, Jung JJ, Curcio CA, Balaratnasingam C, Gallego-Pinazo R, Dolz-Marco R, Freund KB, Yannuzzi LA (2016). Intraretinal Hyperreflective foci in acquired Vitelliform lesions of the macula: clinical and histologic study. Am J Ophthalmol.

[21] Christenbury JG, Folgar FA, O'Connell RV, Chiu SJ, Farsiu S, Toth CA (2013). Progression of intermediate age-related macular degeneration with proliferation and inner retinal migration of hyperreflective foci. Ophthalmology.

[22] Coscas G, De Benedetto U, Coscas F, Li Calzi CI, Vismara S, Roudot-Thoraval F, Bandello F, Souied E (2013). Hyperreflective dots: a new spectral-domain optical coherence tomography entity for follow-up and prognosis in exudative age-related macular degeneration. Ophthalmologica Journal international d'ophtalmologie International journal of ophthalmology Zeitschrift fur Augenheilkunde.

[23] Kuroda M, Hirami Y, Hata M, Mandai M, Takahashi M, Kurimoto Y (2014). Intraretinal hyperreflective foci on spectral-domain optical coherence tomographic images of patients with retinitis pigmentosa. Clin Ophthalmol (Auckland, NZ).

[24] Ogino K, Murakami T, Tsujikawa A, Miyamoto K, Sakamoto A, Ota M, Yoshimura N (2012). Characteristics of optical coherence tomographic hyperreflective foci in retinal vein occlusion. Retina.

[25] Piri N, Nesmith BL, Schaal S (2015). Choroidal hyperreflective foci in Stargardt disease shown by spectral-domain optical coherence tomography imaging: correlation with disease severity. JAMA Ophthalmol.

[26] Murakami T, Yoshimura N (2013). Structural changes in individual retinal layers in diabetic macular edema. J Diabetes Res.

[27] Vujosevic S, Bini S, Midena G, Berton M, Pilotto E, Midena E (2013). Hyperreflective intraretinal spots in diabetics without and with nonproliferative diabetic retinopathy: an in vivo study using spectral domain OCT. J Diabetes Res.

[28] Uji A, Murakami T, Nishijima K, Akagi T, Horii T, Arakawa N, Muraoka Y, Ellabban AA, Yoshimura N (2012). Association between hyperreflective foci in the outer retina, status of photoreceptor layer, and visual acuity in diabetic macular edema. Am J Ophthalmol.

[29] Ehrlich R, Harris A, Ciulla T, Kheradiya N, Winston D, Wirostko B (2010). Diabetic macular oedema: physical, physiological and molecular factors contribute to this pathological process. Acta Ophthalmol.

[30] Kang JW, Lee H, Chung H, Kim HC (2014). Correlation between optical coherence tomographic hyperreflective foci and visual outcomes after intravitreal bevacizumab for macular edema in branch retinal vein occlusion. Graefes Arch Clin Exp Ophthalmol.

[31] Huang J, Li X, Li M, Li S, Xiao W, Chen X, Cai M, Wu Q, Luo D, Tang S (2012). Effects of intravitreal injection of KH902, a vascular endothelial growth factor receptor decoy, on the retinas of streptozotocin-induced diabetic rats. Diabetes Obes Metab.

[32] Ota M, Nishijima K, Sakamoto A, Murakami T, Takayama K, Horii T, Yoshimura N (2010). Optical coherence tomographic evaluation of foveal hard exudates in patients with diabetic maculopathy accompanying macular detachment. Ophthalmology.

